# The Effect of Perceived Supervisor–Subordinate Congruence in Honesty on Emotional Exhaustion: A Polynomial Regression Analysis

**DOI:** 10.3390/ijerph18179420

**Published:** 2021-09-06

**Authors:** Jae-Heon Do, Seung-Wan Kang, Suk Bong Choi

**Affiliations:** 1 Seoul School of Integrated Sciences & Technologies, 46 Ewhayeodae 2-gil, Fintower, Sinchon-ro, Seodaemun-gu, Seoul 03767, Korea; damwoosul@gmail.com; 2 College of Business, Gachon University, 1342 Seongnamdaero, Sujeong-gu, Seongnam-si 13120, Korea; 3 College of Global Business, Korea University, 2511 Sejong-ro, Sejong City 30019, Korea

**Keywords:** honesty, person–supervisor fit, supervisor–subordinate congruence, emotional exhaustion, polynomial regression

## Abstract

Do subordinates unequivocally prefer honest superv =isors? This study investigates whether congruence in supervisor–subordinate honesty has a positive effect on lowering the emotional exhaustion experienced by subordinates. For the research data, a two-part survey with a one-month time interval was conducted among office workers, and 409 responses were collected. These were empirically analyzed using polynomial regression analysis and response surface analysis, instead of the common methodology based on difference values used in past studies on the fit between a person and their environment. The analysis results confirmed that supervisor–subordinate congruence in honesty has a negative relationship with subordinates’ emotional exhaustion and supervisor–subordinate congruence at higher levels of honesty will have stronger negative relationships with emotional exhaustion. This study expands the intrapersonal context of the existing research on supervisors’ honesty to the interpersonal context and empirically demonstrates the effect of honesty congruence. It also discusses its theoretical and practical implications as well as limitations, and it provides suggestions for future studies.

## 1. Introduction

The characteristic of being honest or engaging in honest behavior is widely regarded as a valued and desirable quality in an organization, particularly in the twenty-first century, where business ethics has taken a more central role in organizational research and among business practitioners. Some of the definitions offered by people when explaining honesty include telling the truth, integrity, and respect for property ownership [1]. Honest employees are preferred by managers as they are generally seen as less likely to engage in workplace delinquency [2] and experience lower stress levels and higher job satisfaction [3]. As a result, considerable research has been conducted on the development and fine-tuning of pre-employment techniques of honesty testing for personnel selection [4,5].

While valuable, a personnel selection approach to ensuring employee integrity ignores a key element, specifically the crucial role that supervisors play in whether or not employees follow through on their honest tendencies. First, the unique nature of the supervisor–subordinate relationship is that the former exerts significant influence over the latter, and subordinates model their behaviors and attitudes after their supervisors [6,7,8]. In support of this thesis, prior research has established that even for normally honest employees, it is incumbent on managers to set an ethical tone to ensure that such employees do not engage in dishonest behaviors [9]. Honesty does not exist in a vacuum; individuals high in honesty are still sensitive to their environment. This suggests that a common frame of reference for honesty between a supervisor and subordinate is important to the extent to which employees act on their honest dispositions.

Second, we argue that a misalignment between employees and supervisors with regard to honesty can be a possible source of stress for employees. Zettler et al. [10] noted that while individuals high in honesty are more likely to be cooperative, those low in honesty are more likely to engage in impression management and adapt their behavior strategically to exploit others or benefit from them. Prior research has suggested that dishonest managers create feelings of work alienation, including a sense of powerlessness or lack of job autonomy, meaninglessness, and self-estrangement [11]. For honest subordinates who generally have positive expectations about how others will act [10], navigating the relational dynamics with a dishonest leader could be a source of emotional distress.

Additionally, much of the literature has focused on the potential benefits of honesty [12,13,14] without revealing the other side of the story. Specifically, honesty may not always be a valued trait in certain contexts. For example, Thompson et al. [15] argued that because honesty is strongly associated with reciprocity-related qualities of non-exploitation and fairness [16], they are not only less likely to be perpetrators of exploitation but also more likely to strongly oppose being targets of exploitation and seek equity to balance the scales of justice if they perceive that an injustice has occurred. Thompson and colleagues further argued that honest individuals may seek revenge and engage in retaliatory behaviors through actions such as withholding extra-role behaviors if they feel slighted. This suggests that when working with honest employees, leaders have the additional responsibility of managing employees’ justice perceptions. Thus, when a supervisor–subordinate dyad shows value incongruence with regard to honesty, this could be stressful for both parties, particularly for employees who have less leverage in the dyad’s power dynamics. To address these issues in the honesty literature, this research takes a person-supervisor (P-S) fit approach using the conservation of resources (COR) theory to answer an important question: does supervisor–subordinate value congruence impact subordinate emotional exhaustion?

Members of an organization perceive how well they fit with their organization in terms of the level of fit with the department or organization (person-organization fit), with the supervisor (person–supervisor fit), and with the job (person–job fit), all of which may differ by the individual [17]. Previous studies have found that person–organization fit affects organization-related variables such as organizational commitment and organizational citizenship behavior [18,19], person–supervisor influences variables such as trust in leadership and job stress [20,21], and person–job fit is related to employee attitudes such as job satisfaction, turnover intention, and career success [22,23,24,25,26]. Supervisor–subordinate dyadic relationships carry emotional requirements that may have positive effects (e.g., self-efficacy) or negative effects (e.g., stress and emotional exhaustion) [27]. Research on burnout began by reviewing how employees in the service industry deal with the stress from customer relations [28] but has expanded in scope to examine workplace relationships, such as those with supervisors and colleagues. However, despite the likely influence of supervisor–subordinate personality fit on employee burnout, few studies have investigated this topic using a congruence approach [29].

Along these lines, we use the COR theory to examine the effect of honesty (in)congruence on employees. COR theory is an integrative stress theory that considers both internal and environmental processes. The basic premise of the theory is that individuals strive to obtain, conserve, and protect against the loss of valued resources [30]. One thread within burnout research pertains to how an individual’s lack of resources can trigger burnout. As Demerouti et al. [31] explained, job demands, including the social aspects of the job, require mental effort and thus come with physiological and psychological costs (e.g., exhaustion).

In sum, this research makes several contributions to the literature on honesty and emotional exhaustion. First, this study moves beyond the follower-centric or leader-centric models of honesty to argue that subordinates and supervisors act in tandem to affect employee attitudes. To do this, the study examines honesty congruence using a person–supervisor approach. Second, it highlights that promoting honesty goes beyond hiring honest employees, which requires that organizations foster a conducive environment by hiring honest supervisors and pairing leaders with employees who value honesty to foster and sustain honest behavior. Third, it provides additional insights into the antecedents of emotional exhaustion.

## 2. Theoretical Background and Research Hypotheses

### 2.1. Person–Supervisor Fit

Each member of an organization perceives their levels of fit differently from the organization, supervisor, and job [17]. Numerous studies have been conducted on these different types of fit perception to report the associations between person–organization fit and organization-related variables such as organizational commitment and organizational citizenship behavior [18,19]; person–supervisor fit with employees’ trust in the supervisor and the stress they receive from working under the supervisor [20,21]; and person–job fit with job attitudes, such as job satisfaction, turnover intention, and career success [22,23,24,25,26].

Another aspect of the fit between supervisors and their subordinates is value congruence and similarities in personality. The more similar the supervisor and subordinate are in their personal traits, the easier it will be for them to trust each other. The similarity-attraction hypothesis states that people who are similar in terms of their demographics and personal traits are more attracted to each other [32]; that is, the more similar the individuals are, the more positive they have in their interpersonal relationships. According to Zucker [33], organizational members build trust in their superiors through continuous interaction, and this process is significantly influenced by demographic similarities such as gender, age, educational background, and relational ties (e.g., school and hometown ties). In this line of research, studies have been conducted on supervisor–subordinate value congruence [34], supervisor–subordinate personality similarity [35], and supervisor–subordinate goal congruence [36]. Previous studies on person–supervisor fit have mainly used “positive” variables, such as employee satisfaction, trust, and commitment as dependent variables. Positive variables are generally considered desirable in interpersonal relationships as they contribute to the development and continuation of the relationship. However, research on how these relationships could be maintained and developed by reducing negative variables such as stress and emotional exhaustion could also provide valuable insights.

### 2.2. Honesty Factor and Emotional Exhaustion

Honesty forms a part of the six-dimensional framework for personality structure referred to as the HEXACO model [37,38], and it is represented by the Honesty (H) factor of the model. Common traits associated with this personality include sincerity, loyalty, fair-mindedness, and modesty [39]. Individuals who are high in honesty tend to lean toward cooperation even when there is a clear path to the successful exploitation of the other party [37]. On the other hand, individuals low in honesty are likely to be greedy and exploitative, with a high drive for power and social status. We argue that when supervisors and subordinates hold similar values with regard to honesty, subordinates will feel lower emotional exhaustion because they do not have to worry about how supervisors will receive their honest behaviors.

Emotional exhaustion was originally formulated as a component of the individual stress component of job burnout. However, in recent years, it has been studied as a standalone construct and is often viewed as the main indicator of burnout [40]. It represents the feeling of being overextended and feeling as if one’s emotional and physical resources are depleted [41]. Emotionally exhausted employees are likely to lash out at others by being rude or saying hurtful things; they are less committed to the organization and more likely to partake in counterproductive work behavior, such as arriving to work late, putting minimal effort into tasks, and other employee withdrawal behavior [42]. Other outcomes include attitudinal consequences on job performance, job satisfaction, and intention to leave [43].

Supervisors who are low in honesty tend to believe that the end justifies the means and would not mind refraining from sharing information with the members of the organization because of their intention to monopolize knowledge [44]. Several studies have reported that leaders’ honesty reduces emotional exhaustion among their followers [45,46], while other studies have explored the relationship between a person’s honesty and emotional exhaustion [47]. If honest employees believe that their superiors value honesty, they are more likely to be proactive and take the initiative to report if they perceive something unethical. This also means that honest employees will know that not only their honesty but also their actions may be rewarded. Alternatively, incongruence with regard to honesty could be a source of conflict, and mismatch could be a source of psychological distress for employees. For example, Individuals high in honesty are very cooperative, in the event of low expectation of the interaction partner, cooperation is reduced [48]. Even in a case where the supervisor is high on honesty, and the subordinate is low on honesty, it is the subordinate who is at the end of the power totem and so is disciplined for not acting in accordance with the supervisors’ expectations for honesty.

COR theory defines resources as objects, energies, conditions, or personal characteristics valued in their own right or as a conduit to obtain or preserve other valued resources [49]. The theory is based on the premise that individuals protect and preserve resources that they consider valuable based on their personal traits, working conditions, time availability, passion, and the recognition, support, and reward provided by their supervisors [30,49]. Interpersonal relationships require a significant amount of mental energy as they involve interactions with other people. Interactions with supervisors whose values or personalities do not match with oneself require an even greater use of mental energy, thereby causing the subordinate to undergo considerable stress or negative experiences, such as emotional exhaustion. Therefore, it can be inferred that a subordinate’s emotional exhaustion may be a response mechanism to their relationship and fit with their supervisor rather than an individual personality trait. COR theory asserts that obtaining and retaining social resources creates a sense that people can encounter stressful challenges [50]. Given that social support is a valuable resource for a subordinate, we posit that supervisor–subordinate congruence in honesty is likely to reduce subordinates’ emotional exhaustion. Based on this reasoning, the following hypothesis is posited:

**Hypothesis** **1.** *Supervisor–subordinate congruence in honesty will have a negative relationship with emotional exhaustion. In other words, the higher the supervisors’ and subordinates’ honesty match, the lower the emotional exhaustion of the subordinate*.

We further argue that the impact on emotional exhaustion will be stronger when both a supervisor and subordinate hold similarly high values of honesty, as opposed to similarly low values of honesty. As previously mentioned, honest individuals are loyal and sincere [39]. This means that when both supervisors and subordinates have honest personalities, not only will they trust each other, but the employee’s expectations of the leader doing what is right and not taking advantage of them even in the face of possible exploitation will be met. As honest supervisors are fair-minded, they are likely to give their employees the right credit and reward when they get the work done. Honest individuals do not have a need for power; they consistently deliver and look to contribute to the public good as their contribution is not conditioned on the power of others [51]. This suggests that honest employees will not be threatened by supervisors, and employees may be more comfortable advancing an opposing viewpoint to a supervisor without fear of retaliation.

**Figure 1:**
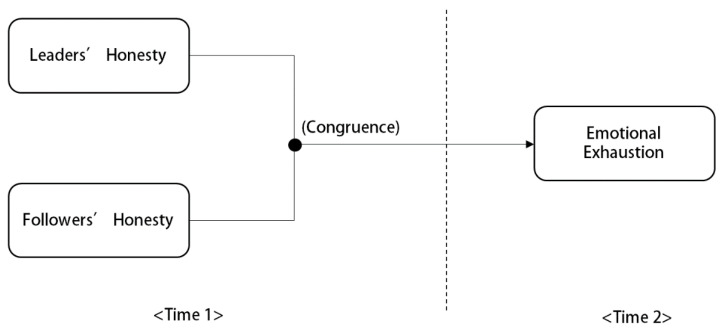
Research Model.

Similarity in the approach to work increases predictability and reduces ambiguity in work-related expectations of one another’s behavior [52]. However, in the case where both parties are dishonest, predictability may not be good. For one, the level of distrust between both parties will be extremely high. If an employee believes that a supervisor is invested in maintaining power and will have no qualms about exploiting a situation to their advantage, this will not cultivate a sense of social support for the employee. COR theory suggests that resource loss is inherently more powerful than resource gain, and it has a stronger effect on people [50]. If an employee cannot trust that a supervisor is genuine in their attention and will not stick up for them if needed, this can be a very stressful scenario for the subordinate. Based on this reasoning, we argue that:

**Hypothesis** **2.** *Supervisor–subordinate congruence at higher levels of honesty will have a stronger negative relationship with emotional exhaustion. In other words, when both the supervisor and subordinate hold high values of honesty, the supervisor–subordinate congruence in honesty will more significantly decrease the subordinates’ emotional exhaustion than when they both hold low values of honesty.The hypothesized research model is illustratedin Figure 1*.

## 3. Research Methodology

### 3.1. Survey Method and Respondent Demographics

The first wave of the survey was conducted in January, 2020, and the second wave of the survey was conducted in February 2020. We recruited target population from a reliable on-line survey company specializing in academic research called Embrain. Target population was randomly selected from an on-line panel consisting of full-time workers in Korea. A two-part survey was conducted among office workers who worked under a supervisor using an online survey tool. According to George and Jones [53], most relational studies on organizational behavior are not immediate, and the changes in one construct are immediately accompanied by changes in another construct. Thus, a time interval of 1 month was placed between the first and second parts of the survey to allow (1) sufficient interaction to take place between the respondents and their supervisors, so that (2) the respondents could observe and experience their supervisors’ decision-making and supervisory behavior for them, and (3) cognitively evaluate those behaviors.

The allocation of the questionnaire was done per the original method outlined by the HEXACO model authors [54]. The original method assessed honesty by having the focal employee evaluate himself/herself and his/her supervisor. Lee and Ashton [54] organized the survey so that participants evaluate themselves and their supervisors. Having subordinates measure their supervisors’ personality traits can prevent potential bias from self-deception, which could arise if supervisors are asked to assess themselves; however, on the other hand, each subordinate may perceive their supervisors’ personality differently. The age and employment status of all respondents were checked to ensure that they met the requirements of the research, and only those who are 20 years old or more and are full-time workers were invited to participate in the survey. Prior to answering the questionnaires, the participants were guided through the purpose and procedures of the research, their freedom to withdraw from the survey at any time, and the benefits and disadvantages that may arise from participating in this research; they were then asked to sign an informed consent form. Data were collected only from those who signed the consent form. The respondents were given a small compensation (USD 3.00 per survey for the two surveys, making a total of USD 6.00) from the online survey platform for their participation.

The first wave of the survey, which contained items measuring the respondents’ and their supervisors’ honesty (independent variable), was sent to 2551 individuals via e-mail. Six hundred responses were collected as an outcome, and after excluding 66 unreliable responses (including 35 incomplete responses), a total of 534 responses were obtained. After 4 weeks, the second wave of the survey containing items on job burnout (dependent variable) was sent to the respondents who completed the first survey via e-mail; of the 438 respondents who completed the second questionnaire, after excluding 29 unreliable responses (including 21 incomplete ones), a total of 409 responses were collected (response rate: 76.6%).

The demographics of the respondents were as follows: 48.9% were male and 51.1% were female, showing an almost equal gender composition. The mean age of the respondents was 37.8 years (SD = 7.75), and 76.8% were in their 30 s and 40 s. The highest level of education completed by the respondents was in the order of bachelor’s degree (62.4%), junior college degree (21.3%), high school diploma (10.8%), master’s degree (4.2%), and doctoral degree (1.5%). In terms of whether they were in supervisory positions, 36.9% were managers, and 63.1% were not. A total of 31.8% were entry-level employees, 26.4% were assistant managers, 22.3% were managers, 9.1% were deputy directors, 8.1% were directors, and 2.4% were executives. The average tenure at their current job was 6 years and 8 months (SD = 5.95).

### 3.2. Measurement Items

The questionnaire items of the research variables were measured using a five-point Likert scale (1 being the lowest score, and 5 being the highest). The original questionnaire items were in English; they were then back-translated into Korean [55] and then reviewed and revised by a bilingual (English–Korean) expert. The resultant questionnaire in Korean was reverse-translated into English to compare the similarities in linguistic structure and meaning with the original text, with the aim to ensure the validity of the translation. A full questionnaire is provided in Appendix A.

#### 3.2.1. Honesty

Following Lee et al. [56], honesty was measured using 10 items asking the respondents to evaluate themselves and 10 items to evaluate their supervisors (20 items in total). Ashton and Lee [39] defined honesty as “the tendency to be fair and genuine in dealing with others, in the sense of cooperation with others even when one might exploit others without suffering retaliation. Some examples of the items measuring the respondents’ honesty were: “Making a lot of money is not a high priority in my life” and “I would not engage in flattering my supervisor even if it helped me get a promotion or a raise.” Using the same method, the respondents were asked to assess how similar their own honesty and the perceived honesty of their supervisors were in terms of the question items. The supervisors’ honesty was measured based on how it is perceived from the standpoint of the subordinate, following a previous study that measured the supervisors’ ability, benevolence, and integrity based on the subordinates’ perceptions and evaluations [57]. Examples of the items on the perceived honesty of the supervisor were: “My supervisor would think that making a lot of money is not a high priority in their life,” and “My supervisor would not engage in flattering their supervisor even if it helped them get a promotion or a raise.” The reliability of the measurement tool for honesty was confirmed, and the inter-item consistency (Cronbach’s alpha) was 0.68 for the respondent (subordinate) honesty and 0.79 for supervisor honesty, which is considered adequate.

#### 3.2.2. Emotional Exhaustion

Emotional exhaustion was measured using five items developed by Kalliath et al. [58]. Examples of the questionnaire items included: “I feel emotionally exhausted from doing my job,” and “I feel completely exhausted on my way back home from work.” The reliability and inter-item consistency (α = 0.91) of the questionnaire items were found to be suitable.

#### 3.2.3. Control Variables

Among demographic characteristics, studies on the factors influencing emotional exhaustion present conflicting arguments on the effect of gender. One study suggested that there are gender differences in the experience of emotional exhaustion. Several studies have also shown that this may be affected by work experience, age, and job tenure. In the case of the number of years of service, prior studies have found that work experience (including job tenure) and age are associated with job burnout [59,60,61,62]. Since the findings of previous studies differ depending on the subject and characteristics of research, gender, job tenure, and age were set as control variables for a more refined analysis. Gender was treated as a binary variable, age was expressed in years, and job tenure was expressed in months.

### 3.3. Analysis Method

Edwards et al. stated that when testing the effect of fit in organizational behavior research, the common methodology using difference values is inherently vague, confuses the characteristics of components, and implies a set of constraints that are rarely tested [63]. Therefore, this study analyzed the data and tested the two hypotheses using polynomial regression; this is a special form of multiple regression analysis that handles the interactions between two different types of explanatory variables by including the power of each variable when the dataset shows a nonlinear relationship. Polynomial regression was performed following the procedure established by Edwards and Rothbard [64] and used by Cole, Carter, and Zhang as well as by Cao and Hamori [65,66].

First, since response surface analysis is sensitive to influential cases [67], the variables were standardized to facilitate the interpretation of the results and avoid the problem of multicollinearity. Regression analysis was performed on the control variables and five polynomial terms: the leaders’ honesty (L), the followers’ honesty (F), the leaders’ honesty squared (L^2^), the followers’ honesty squared (F^2^), and the product of the leaders’ and followers’ honesty values (L × F). The results of the polynomial regression were plotted on a three-dimensional response surface graph. Lastly, the slope and curvature, which are the most important components of response surface analysis, were calculated based on L = F (line of numerical congruence, LOC) and L = -F (line of numerical incongruence, LOIC). The curvature of the response surface on the LOIC was verified to check whether the subordinate experienced less emotional exhaustion when their value of honesty was congruent with that of their supervisor. Here, a statistically significant positive curvature denotes that supervisor–subordinate congruence in honesty has a negative relationship with emotional exhaustion (Hypothesis 1). The slope of the LOC was also verified to compare how the level of emotional exhaustion changes when the supervisor and subordinate both value honesty at high levels as opposed to when they both value honesty at low levels. A statistically significant negative slope means that supervisor–subordinate congruence at higher levels of honesty is more effective in lowering subordinates’ emotional exhaustion than when congruence is formed at lower levels of honesty (Hypothesis 2).

## 4. Results

We conducted a CFA to assess the discriminant validity of our study measures. Table 1 depicts the results of the CFA, the results of model fit regarding Leaders’ Honesty, Followers’ Honesty, and Emotional exhaustion. First, the absolute fit index(χ^2^ / *df*) was 1.73 (χ^2^ = 405.84, *df* = 234), which was less than the cutoff value of 3.00 [68]. Second, the incremental fit indices, the comparative fit index (CFI), and Tucker–Lewis index (TLI) values were 0.95 and 0.94, which exceeded the standard cutoff values of 0.90. In addition, the root mean square error of approximation was 0.04, which was less than the standard cutoff value of 0.08 [69]. All of the CFA indicators satisfied the standards verification by which we determined that our hypothesized measurement model was appropriate for the data.

Table 2 shows the mean, standard deviation, and correlation of all variables. The mean of subordinates’ honesty is higher than that of supervisors’ honesty, which shows that, overall, the respondents evaluated themselves more generously than their supervisors.

**Figure 2:**
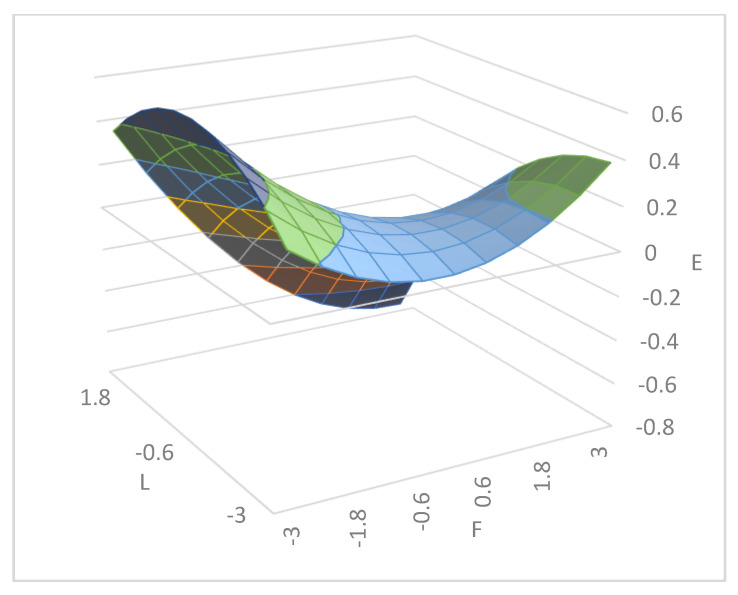
The effect of supervisor–subordinate congruence in honesty on the subordinates’ emotional exhaustion. Note: L = Leaders’ honesty; F = Followers’ honesty; E = Emotional exhaustion.

**Figure 3:**
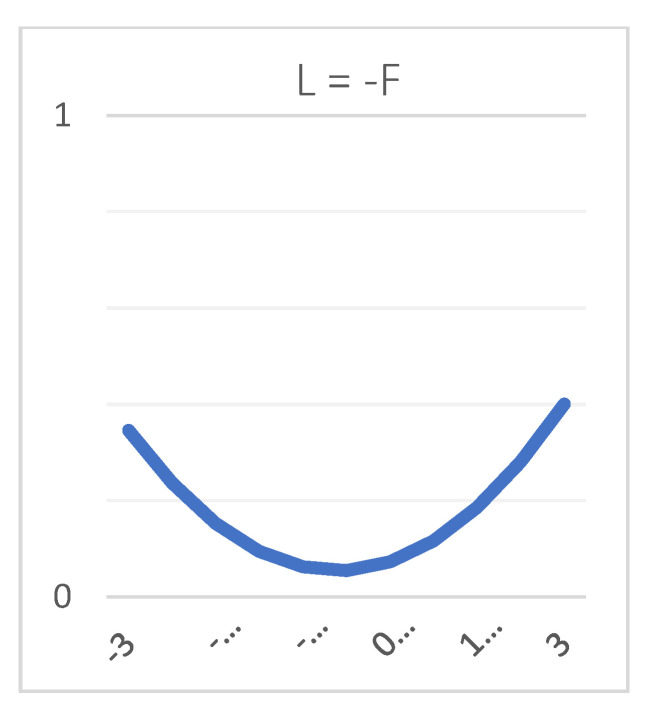
Curvature of the line of numerical incongruence (L = −F). Note: L = Leaders’ honesty; F = Followers’ honesty, x-axis= incongruence line.

Table 3 shows the polynomial regression coefficients representing the slope and curvature along LOC and LOIC of the response surface, and Figure 2 presents the three-dimensional graph of the response surface. For Hypothesis 1 to be supported, two conditions must be satisfied: (a) the effect of the quadratic polynomial terms (F2, F × L, L2) must have a significant F value, and (b) the curvature along the LOIC should be significant in the positive direction [63]. Compared to Model 1, Model 2 of Table 3 shows an increase in R2 (ΔR2 = 0.01), with F = 2.03, *p* < 0.01, and the LOIC forms a significant upward curve as shown in Figure 3 (Curvature = 0.04, *p* < 0.01). These results confirm that the congruence in supervisor–subordinate honesty lowers the subordinates’ emotional exhaustion, which also suggests that incongruence in supervisor–subordinate honesty is associated with high emotional exhaustion; therefore, Hypothesis 1 is supported.

**Figure 4:**
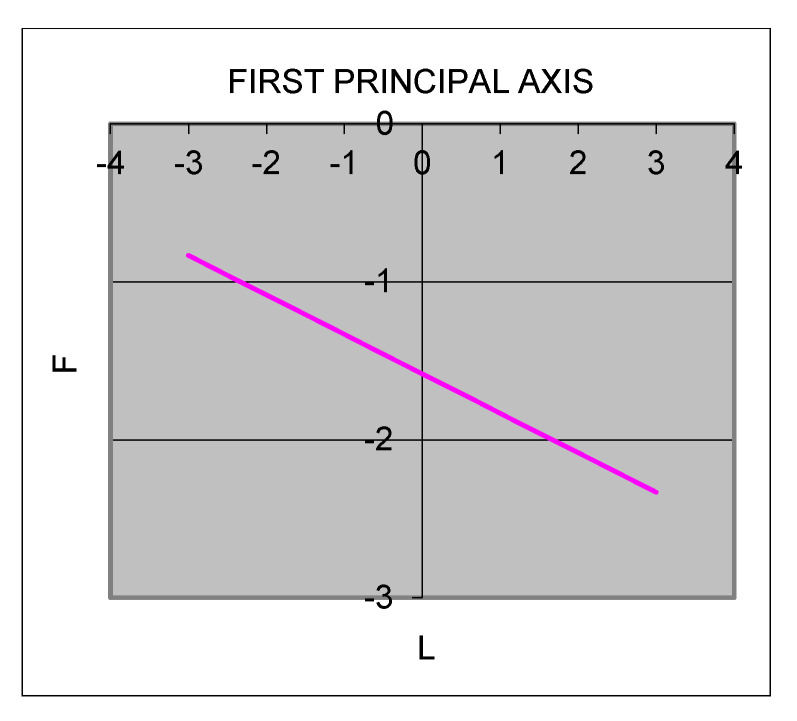
Slope of the line of numerical congruence (L = F). Note: L = Leaders’ honesty; F = Followers’ honesty.

For Hypothesis 2 to be true, LOC must have a significant negative slope. The slope of the LOC in Model 2 of Table 3 is significantly negative (slope = −0.02, *p* < 0.001), as visualized in Figure 4, confirming that even when the supervisors’ and subordinates’ values of honesty match, congruence at a higher level lowers the subordinates’ emotional exhaustion to a greater degree than value congruence at a lower level. Moreover, from the shape of the response surface, shown in Figure 2, we can see that the subordinates’ emotional exhaustion is lower in the rear corner than in the front corner, which shows that the results of the response surface analysis are consistent with those of statistical verification. Therefore, Hypothesis 2 is supported.

## 5. Discussion

### 5.1. Theoretical Contribution

This study contributes to the academic literature on organizational behavior in the following ways. First, although a number of studies have explored person–supervisor fit in terms of similarities in traits, personality congruence, and regulatory fit [70], this is the first empirical study that specifically investigated supervisor–subordinate congruence in honesty. It sought to verify the joint effect of supervisors’ and subordinates’ values of honesty as an antecedent of employee emotional exhaustion. To this end, the congruence of the supervisors’ and subordinates’ values of honesty was examined and analyzed using polynomial regression analysis. The findings of this study provide several insights. First, the congruence in supervisor–subordinate honesty showed a negative relationship with emotional exhaustion; in other words, we showed that the greater the congruence in supervisor–subordinate honesty, the lower the subordinates’ emotional exhaustion. We also showed that the supervisor–subordinate congruence in honesty will have a greater effect on reducing subordinates’ emotional exhaustion when both parties value honesty at higher levels than at low levels. When the leader has a high level of honesty, they will require followers to conform to higher standards, which may be difficult for subordinates with low levels of honesty and cause them to experience greater emotional exhaustion for fear that they will lose resources, such as the recognition and trust of their leader.

Second, from the standpoint of research methodology, this study presents a useful example of how polynomial regression and response surface analysis can be used to determine how the congruence or incongruence of the independent variables (supervisors’ honesty and subordinates’ honesty) affects the dependent variable (subordinates’ emotional exhaustion). When supervisor–subordinate honesty was incongruent, the relationship between the supervisors’ honesty and the subordinates’ emotional exhaustion formed a U-shaped curve, which empirically confirmed the importance of both the supervisors’ and the subordinates’ honesty.

Third, this study helps fill the gap in the research on the relationship between supervisors’ honesty and subordinates’ emotional exhaustion. Existing studies on the antecedents of emotional exhaustion have mainly focused on situational or organizational factors, such as the discrepancy between the job competency required by the organization and that of the organizational member, or on individual-level characteristics, such as differences in the responses and job stress when facing the abusive behavior of supervisors by gender and personality traits [71]. Another stream of research provides a one-sided understanding of how the dark sides of leaders’ personalities affect their followers’ trust and emotional exhaustion. This study expands on existing perspectives concentrating on the situational, organizational, or one-way factors that cause emotional exhaustion by introducing a viewpoint from the interpersonal context of the dyadic relationship that subordinates have with their supervisors and how emotional exhaustion could arise from the differences in personality (and especially in the honesty) perceived through this relationship.

### 5.2. Practical Implications

The increasingly unpredictable changes in the business environment have underscored the importance of securing, maintaining, and developing human resources with outstanding capabilities. Fundamentally, business ethics are virtues that firms must pursue at all times, which can only be practiced regularly when each member of the firm prioritizes them. This study has several practical implications. First, research showing the benefits of hiring honest employees is well established; consequently, firms invest substantially in pre-employment techniques of honesty testing for personnel selection [4,5]. However, this research suggests that even when firms are able to hire the most honest employees, the right contexts and conditions must be created to sustain and encourage employees to act on their honest tendencies. This study suggests that personality congruence with supervisors regarding honesty provides the right context to sustain the practice of honesty in firms. Beyond honesty, firms may need to consider pairing supervisors with subordinates with similar personality traits.

Second, previous studies have focused mainly on the one-sided effect of leaders’ personality traits on followers’ emotional exhaustion. In contrast, this study considers the honesty of both the supervisor and subordinate to identify how the congruence or incongruence in their values of honesty affects emotional exhaustion using polynomial regression analysis and response surface analysis. That is, this study deals with emotional exhaustion not as an intrapersonal outcome based on individual personality traits but as a dimension of interpersonal dyadic relationships. By doing so, the findings reaffirm the need for healthy supervisor–subordinate relationships and for concerted efforts to help organizational members form healthy relationships and build a good organizational culture.

Lastly, organizations must consider the possible negative effect of supervisor–subordinate honesty incongruence on employees. In a situation where an honest supervisor is paired with a subordinate who does not hold similarly high values of honesty, this can be a source of psychological distress for the latter. Given that between a supervisor and subordinate, the power dynamics favor the subordinate, the employee is the one who would feel the fuller brunt of emotional exhaustion. This could have larger implications for employee job satisfaction, job performance, and intention to leave. Organizations must acknowledge that incongruence in supervisor–subordinate honesty could hinder employee acclimatization and growth, thus requiring proactive preventive measures. When assigning personnel to tasks that are highly sensitive and require the highest ethical standards, organizations will be well served not just by simply selecting an honest supervisor or honest employees but rather by pairing supervisor–subordinate dyads that are congruent in their high honesty values to elicit honest behavior.

### 5.3. Limitations and Suggestions for Future Research

Despite its contributions, this study has several limitations. First, this study was surveyed Korean office workers only, who may share specific cultural characteristics and lend to a specific interpretation of the questionnaire. Thus, to generalize the findings of this research requires supplementation through subsequent research.

Second, the survey was based on a self-report questionnaire, which inherently carries the risk of common method bias. To mitigate this problem, the survey was conducted in two parts with a 1-month interval between the two questionnaires containing the measurement items for the independent variable and the dependent variables, respectively, as proposed by Podsakoff, MacKenzie [72]. However, it may be necessary to set longer time intervals. Additionally, a different survey method may be applied; for example, supervisors could assess their subordinates’ honesty and vice versa.

Third, including other variables (such as narcissism, extraversion) used in existing research may deliver more refined results on the degree to which supervisor–subordinate congruence in honesty affects the subordinates’ emotional exhaustion as an antecedent. For instance, setting the critical antecedent identified in previous literature as control variables may reveal further insights into the relationship between supervisor–subordinate honesty and emotional exhaustion. For occupations in law enforcement, accounting, compliance, or involving saving a person’s life, a relatively higher honesty is required compared to other occupations. Therefore, it would be meaningful to conduct additional research using occupational characteristics as a control variable or to compare the results by dividing the occupational groups that required relatively higher honesty and those that are do not. Furthermore, if the supervisor follows a stricter standard of honesty than the subordinate, this does not necessarily mean that the supervisor should lower their standard to match that of the subordinate. Further studies are needed on how supervisors should handle subordinates with lower honesty when it is essential that the former maintain high honesty. This aspect could be explored in future research on moderating variables between supervisor–subordinate honesty and emotional exhaustion.

Finally, this study examined emotional exhaustion among the three burnout components for two reasons. First, emotional exhaustion is considered the most critical component of burnout, and second, emotional exhaustion sequentially affects the other two components, namely dehumanization and a diminished sense of achievement [73,74]. Previous studies have also focused on emotional exhaustion as a representative component of burnout [75,76]. Thus, future studies may explore the other two components—dehumanization and a diminished sense of achievement—to gain a deeper understanding of the causes and factors of job burnout.

## Figures and Tables

**Table 1:** Results of Confirmatory Factor Analysis.

Measurement Model	χ^2^	*df*	*p*-Value	CFI	TLI	RMSEA
Three factor model	405.84	234	0.000	0.95	0.94	0.04

Note. *n* = 325; CFI = comparative fit index; TLI = Tucker–Lewis index; RMSEA = root mean square error of approximation.

**Table 2:** Mean, Standard Deviation, and Correlations.

Variable	Mean	SD	1	2	3	4	5	6
1. Age	37.81	7.75						
2. Gender	0.50	0.50	0.36 ***					
3. Job tenure	6.67	5.95	0.58 ***	0.26 ***				
4. Leaders’ honesty	3.04	0.64	−0.03	0.05	−0.06	(0.79)		
5. Followers’ honesty	3.46	0.51	0.09	−0.05	0.11 *	0.25 ***	(0.68)	
6. Emotional exhaustion	3.14	0.90	−0.11 *	−0.07	−0.07	−0.12 *	−0.11 *	(0.91)

Note. *n* = 409. * *p* < 0.05, *** *p* < 0.001, the values in parentheses denote inter-item consistency, Age = year, Job tenure = year, Gender: Female = 0, Male = 1.

**Table 3:** Polynomial regression on the effect of supervisor–subordinate congruence in honesty on emotional exhaustion.

	Emotional Exhaustion	
Variable	Model 1	Model 2
Age	−0.08 (0.06)	−0.08 (0.06)
Gender	−0.08 (0.11)	−0.10 (0.11)
Job tenure	−0.01 (0.06)	−0.01 (0.06)
Leaders’ honesty (L)	−0.09 (0.05)	−0.09 (0.05)
Followers’ honesty (F)	−0.09 (0.05)	−0.08 (0.05)
L^2^		−0.03 (0.04)
L × F		−0.03 (0.05)
F^2^		0.03 (0.04)
R^2^	0.03	0.04
ΔR^2^		0.01
F for the three quadratic terms		2.03 *
LOC (L = F)		
Slope		−0.02 ***
Curvature		−0.03
LOIC (L = −F)		
Slope		0.01
Curvature		0.04 ***

Note. Value of the non-standardized regression coefficient; the numbers in parentheses indicate standard error. * *p* < 0.05, *** *p* < 0.001

## Data Availability

Not applicable.

## Appendix A

Subordinate honesty (Cronbach’s alpha = 0.68) [56].

1. I would not engage in flattering my supervisor even if it helped me get a promotion or a raise.
2. I would steal a few ten million won if I was sure I would not get caught.
3. Making a lot of money is not a high priority in my life.
4. I feel that I deserve more respect than the average person.
5. I try to please people, even if I hate it, if I need something from them.
6. I do not accept bribes, however big or small.
7. I wish to own expensive luxury goods.
8. I want others to treat me like an important, high-status person.
9. I do not pretend to like someone in order to ask them a favor.
10. I would be tempted to use counterfeit bills as long as it’s guaranteed that I will not be caught.

Supervisor honesty (Cronbach’s alpha = 0.79)

1. My supervisor would not engage in flattering his/her supervisor even if it helped him/her get a promotion or a raise.
2. My supervisor would steal a few 10 million won if he/she was sure he/she would not get caught.
3. My supervisor would think that making a lot of money is not a high priority in his/her life.
4. My supervisor feels that he/she deserves more respect than the average person.
5. My supervisor tries to please people, even if he/she hates it, if he/she needs something from them.
6. My supervisor does not accept bribes, however big or small.
7. My supervisor wishes to own expensive luxury goods.
8. My supervisor wants others to treat him/her like an important, high-status person.
9. My supervisor does not pretend to like someone in order to ask them a favor.
10. My supervisor would be tempted to use counterfeit bills as long as it’s guaranteed that he/she will not be caught.

Emotional exhaustion (Cronbach’s alpha = 0.91) [58].

1. I feel emotionally drained from my work.
2. I feel fatigued when I get up in the morning and have to face another day on the job.
3. I feel frustrated by my job.
4. I feel I’m working too hard on my job.
5. I feel like I’m at the end of my rope.

## References

[1] Elm D.R., Giacalone R.A., Jurkiewicz C.L. (2003). Honesty, spirituality, and performance at work. Handbook of Workplace Spirituality and Organizational Performance.

[2] De Vries R.E., Van Gelder J.L. (2015). Explaining workplace delinquency: The role of Honesty–Humility, ethical culture, and employee surveillance. Personal. Individ. Differ..

[3] Wiltshire J., Bourdage J.S., Lee K. (2014). Honesty-humility and perceptions of organizational politics in predicting workplace outcomes. J. Bus. Psychol..

[4] Sackett P.R., Wanek J.E. (1996). New developments in the use of measures of honesty integrity, conscientiousness, dependability trustworthiness, and reliability for personnel selection. Pers. Psychol..

[5] Morgeson F.P., Campion M.A., Dipboye R.L., Hollenbeck J.R., Murphy K., Schmitt N. (2007). Reconsidering the use of personality tests in personnel selection contexts. Pers. Psychol..

[6] Engle E.M., Lord R.G. (1997). Implicit theories, self-schemas, and leader-member exchange. Acad. Manag. J..

[7] Graen G.B., Scandura T.A. (1987). Toward a Psychology of Dyadic Organizing. Res. Organ. Behav..

[8] Opoku M.A., Choi S.B., Kang S.-W. (2019). Servant Leadership and Innovative Behaviour: An Empirical Analysis of Ghana’s Manufacturing Sector. Sustainability.

[9] Litzky B.E., Eddleston K.A., Kidder D.L. (2006). The good, the bad, and the misguided: How managers inadvertently encourage deviant behaviors. Acad. Manag. Perspect..

[10] Zettler I., Hilbig B.E., Heydasch T. (2013). Two sides of one coin: Honesty-Humility and situational factors mutually shape social dilemma decision making. J. Res. Personal..

[11] Zoghbi-Manrique-de-Lara P., Viera-Armas M. (2019). Using Alienation at Work to Explain Why Managers’ Dishonesty Does Not Lead to Firm Performance. Eur. Manag. Rev..

[12] Kirkland J.C. (2019). Effect of Honest and Humble Leadership on Sales Outcome. Ph.D. Dissertation.

[13] Anand A., Walsh I., Moffett S. (2019). Does humility facilitate knowledge sharing? Investigating the role of humble knowledge inquiry and response. J. Knowl. Manag..

[14] Lee Y., Berry C.M., Gonzalez-Mulé E. (2019). The importance of being humble: A meta-analysis and incremental validity analysis of the relationship between honesty-humility and job performance. J. Appl. Psychol..

[15] Thompson M., Carlson D., Hunter E., Whitten D. (2016). We all seek revenge: The role of honesty-humility in reactions to incivility. J. Behav. Appl. Manag..

[16] Ashton M.C., Lee K. (2001). A theoretical basis for the major dimensions of personality. Eur. J. Personal..

[17] Kristof-Brown A.L., Zimmerman R.D., Johnson E.C. (2005). Consequences of Individuals’ fit at Work: A Meta-Analysis of Person-Job, Person-Organization, Person-Group, and Person-Supervisor Fit. Pers. Psychol..

[18] Goodman S.A., Svyantek D.J. (1999). Person–organization fit and contextual performance: Do shared values matter. J. Vocat. Behav..

[19] Silva N., Thoman A., Mayoral L., Yoshida M. Organizational Strategy and Employee Outcomes. Proceedings of the 17th Annual Conference of the Society of Industrial and Organizational Psychology.

[20] Choi M.O., Yoo T.Y. (2005). The Effects of Person-Organization, Person-Job, and Person-Supervisor Fit on Organization Commitment, Job Satisfaction, and Turnover Intention: The Focus on Interaction Effects among Three Types of Fit. Korean J. Ind. Organ. Psychol..

[21] Ravlin E.C., Ritchie C.M. (2006). Perceived and actual organizational fit: Multiple influences on attitudes. J. Manag. Issues.

[22] Yoo T.-Y., Hyun H.-J. (2003). Effects of the fit of current-ideal organizational personality and the fit of current-ideal job characteristics on the attitudes toward the organization and the job. Korean Psychol. Assoc..

[23] Autry C.W., Daugherty P.J. (2003). Warehouse operations employees: Linking person-organization fit, job satisfaction, and coping responses. J. Bus. Logist..

[24] Erdogan B., Kraimer M.L., Liden R.C. (2004). Work value congruence and intrinsic career success: The compensatory roles of leader-member exchange and perceived organizational support. Pers. Psychol..

[25] Lauver K.J., Kristof-Brown A. (2001). Distinguishing between employees’ perceptions of person-job and person-organization fit. J. Vocat. Behav..

[26] Vigoda E., Cohen A. (2002). Influence tactics and perceptions of organizational politics: A longitudinal study. J. Bus. Res..

[27] Ortiz-Bonnín S., García-Buades M.E., Caballer A., Zapf D. (2016). Supportive climate and its protective role in the emotion rule dissonance-emotional exhaustion relationship: A multilevel analysis. J. Pers. Psychol..

[28] Cordes C.L., Dougherty T.W. (1993). A review and an integration of research on job burnout. Acad. Manag. Rev..

[29] Wesolowski M.A., Mossholder K.W. (1997). Relational demography in supervisor-subordinate dyads: Impact on subordinate job satisfaction, burnout, and perceived procedural justice. J. Organ. Behav. Int. J. Ind. Occup. Organ. Psychol. Behav..

[30] Hobfoll S.E. (1989). Conservation of resources: A new attempt at conceptualizing stress. Am. Psychol..

[31] Demerouti E., Bakker A.B., Nachreiner F., Schaufeli W.B. (2001). The job demands-resources model of burnout. J. Appl. Psychol..

[32] Byrne D.E. (1971). The Attraction Paradigm.

[33] Zucker L.G. (1986). Production of trust: Institutional sources of economic structure, 1840–1920. Res. Organ. Behav..

[34] Colbert A.E., Mount M.K., Harter J.K., Witt L.A., Barrick M.R. (2004). Interactive effects of personality and perceptions of the work situation on workplace deviance. J. Appl. Psychol..

[35] Schaubroeck J., Lam S.S. (2002). How similarity to peers and supervisor influences organizational advancement in different cultures. Acad. Manag. J..

[36] Witt L. (1998). Enhancing organizational goal congruence: A solution to organizational politics. J. Appl. Psychol..

[37] Ashton M.C., Lee K., De Vries R.E. (2014). The HEXACO Honesty-Humility, Agreeableness, and Emotionality factors: A review of research and theory. Personal. Soc. Psychol. Rev..

[38] Lee K., Ashton M.C. (2004). Psychometric properties of the HEXACO personality inventory. Multivar. Behav. Res..

[39] Ashton M.C., Lee K. (2007). Empirical, theoretical, and practical advantages of the HEXACO model of personality structure. Personal. Soc. Psychol. Rev..

[40] Cropanzano R., Rupp D.E., Byrne Z.S. (2003). The relationship of emotional exhaustion to work attitudes, job performance, and organizational citizenship behaviors. J. Appl. Psychol..

[41] Maslach C., Schaufeli W.B., Leiter M.P. (2001). Job burnout. Annu. Rev. Psychol..

[42] Bennett R.J., Robinson S.L. (2000). Development of a measure of workplace deviance. J. Appl. Psychol..

[43] Edmondson D.R., Matthews L.M., Ambrose S.C. (2019). A meta-analytic review of emotional exhaustion in a sales context. J. Pers. Sell. Sales Manag..

[44] Kang S.-W., Kim N. (2019). An Experimental Study on the Influence of Knowledge Psychological Ownership on Knowledge Withholding Intention-Moderating Effect of Machiavellianism Personality. J. Hum. Resour. Manag. Res..

[45] Gkorezis P., Petridou E., Krouklidou T. (2015). The detrimental effect of machiavellian leadership on employees’ emotional exhaustion: Organizational cynicism as a mediator. Eur. J. Psychol..

[46] Stradovnik K., Stare J. (2018). Correlation between Machiavellian leadership and emotional exhaustion of employees. Leadersh. Organ. Dev. J..

[47] Zhao J., Xiao S., Mao J., Liu W. (2018). The buffering effect of Machiavellianism on the relationship between role conflict and counterproductive work behavior. Front. Psychol..

[48] Pfattheicher S., Böhm R. (2018). Honesty-humility under threat: Self-uncertainty destroys trust among the nice guys. J. Personal. Soc. Psychol..

[49] Hobfoll S.E. (2001). The influence of culture, community, and the nested-self in the stress process: Advancing conservation of resources theory. Appl. Psychol..

[50] Hobfoll S.E., Halbesleben J., Neveu J.P., Westman M. (2018). Conservation of resources in the organizational context: The reality of resources and their consequences. Annu. Rev. Organ. Psychol. Organ. Behav..

[51] Hilbig B.E., Zettler I., Heydasch T. (2012). Personality, punishment and public goods: Strategic shifts towards cooperation as a matter of dispositional honesty-humility. Eur. J. Personal..

[52] Zhang Z., Wang M.O., Shi J. (2012). Leader-follower congruence in proactive personality and work outcomes: The mediating role of leader-member exchange. Acad. Manag. J..

[53] George J.M., Jones G.R. (2000). The role of time in theory and theory building. J. Manag..

[54] Lee K., Ashton M.C. (2013). The H Factor of Personality: Why Some People Are Manipulative, Self-Entitled, Materialistic, and Exploitive—And Why It Matters for Everyone.

[55] Brislin R.W. (1980). Translation and content analysis of oral and written materials. Handbook of Cross-Cultural Psychology: Methodology.

[56] Lee K., Ogunfowora B., Ashton M.C. (2005). Personality traits beyond the Big Five: Are they within the HEXACO space?. J. Personal..

[57] Colquitt J.A., Rodell J.B. (2011). Justice, trust, and trustworthiness: A longitudinal analysis integrating three theoretical perspectives. Acad. Manag. J..

[58] Kalliath T.J., O’Driscoll M.P., Gillespie D.F., Bluedorn A.C. (2000). A test of the Maslach Burnout Inventory in three samples of healthcare professionals. Work Stress.

[59] Caplan R.D., Jones K.W. (1975). Effects of work load, role ambiguity, and type A personality on anxiety, depression, and heart rate. J. Appl. Psychol..

[60] Chapman D.W., Lowther M.A. (1982). Teachers’ satisfaction with teaching. J. Educ. Res..

[61] Anderson M.B.G., Iwanicki E.F. (1984). Teacher motivation and its relationship to burnout. Educ. Adm. Q..

[62] Arricale F. (2001). A Study of Burnout of Counselors in College Counseling Centers.

[63] Edwards J.R., Parry M.E. (1993). On the use of polynomial regression equations as an alternative to difference scores in organizational research. Acad. Manag. J..

[64] Edwards J.R., Rothbard N.P. (1999). Work and family stress and well-being: An examination of person-environment fit in the work and family domains. Organ. Behav. Hum. Decis. Process..

[65] Cole M.S., Carter M.Z., Zhang Z. (2013). Leader-team congruence in power distance values and team effectiveness: The mediating role of procedural justice climate. J. Appl. Psychol..

[66] Cao J., Hamori M. (2020). How can employers benefit most from developmental job experiences? The needs-supplies fit perspective. J. Appl. Psychol..

[67] Edwards J.R., Cable D.M. (2009). The value of value congruence. J. Appl. Psychol..

[68] Hair J., Black W., Anderson R. (2010). Multivariate Data Analysis: A Global Perspectives.

[69] Hu L.T., Bentler P.M. (1999). Cutoff criteria for fit indexes in covariance structure analysis: Conventional criteria versus new alternatives. Struct. Equ. Modeling Multidiscip. J..

[70] Shin Y., Kim M.S., Choi J.N., Kim M., Oh W.-K. (2017). Does leader-follower regulatory fit matter? The role of regulatory fit in followers’ organizational citizenship behavior. J. Manag..

[71] Kim N., Kang Y.J., Choi J., Sohn Y.W. (2020). The Crossover Effects of Supervisors’ Workaholism on Subordinates’ Turnover Intention: The Mediating Role of Two Types of Job Demands and Emotional Exhaustion. Int. J. Environ. Res. Public Health.

[72] Podsakoff P.M., MacKenzie S.B., Lee J.-Y., Podsakoff N.P. (2003). Common method biases in behavioral research: A critical review of the literature and recommended remedies. J. Appl. Psychol..

[73] Maslach C. (1982). Understanding burnout: Definitional issues in analyzing a complex phenomenon. Job Stress and Burnout.

[74] Baba V.V., Jamal M., Tourigny L. (1998). Work and mental health: A decade in Canadian research. Can. Psychol. Psychol. Can..

[75] Leiter M.P. (1991). Coping patterns as predictors of burnout: The function of control and escapist coping patterns. J. Organ. Behav..

[76] Babakus E., Cravens D.W., Grant K., Ingram T.N., LaForge R.W. (1996). Investigating the relationships among sales, management control, sales territory design, salesperson performance, and sales organization effectiveness. Int. J. Res. Mark..

